# New analytic approaches for analyzing and presenting polio surveillance data to supplement standard performance indicators

**DOI:** 10.1016/j.jvacx.2020.100059

**Published:** 2020-03-21

**Authors:** Kristin VanderEnde, Arend Voorman, Sara Khan, Abhijeet Anand, Cynthia J Snider, Ajay Goel, Steve Wassilak

**Affiliations:** a Centers for Disease Control and Prevention, Atlanta 30329, USA; b The Bill and Melinda Gates Foundation, Seattle 98109, USA; c The World Health Organization, Geneva 1202, Switzerland

**Keywords:** Poliomyelitis, Disease surveillance, Data quality

## Abstract

**Background:**

Sensitive surveillance for acute flaccid paralysis (AFP) allows for rapid detection of polio outbreaks and provides essential evidence to support certification of the eradication of polio. However, accurately assessing the sensitivity of surveillance systems can be difficult due to limitations in the reliability of available performance indicators, including the rate of detection of non-polio AFP and the proportion of adequate stool sample collection. Recent field reviews have found evidence of surveillance gaps despite indicators meeting expected targets.

**Methods:**

We propose two simple new approaches for AFP surveillance performance indicator analysis to supplement standard indicator analysis approaches commonly used by the Global Polio Eradication Initiative (GPEI): (1) using alternative groupings of low population districts in the country (spatial binning) and (2) flagging unusual patterns in surveillance data (surveillance flags analysis). Using GPEI data, we systematically compare AFP surveillance performance using standard indicator analysis and these new approaches.

**Results:**

Applying spatial binning highlights areas meeting surveillance indicator targets that do not when analyzing performance of low population districts. Applying the surveillance flags we find several countries with unusual data patterns, in particular age groups which are not well-covered by the surveillance system, and countries with implausible rates of adequate stool specimen collection.

**Conclusions:**

Analyzing alternate groupings of administrative units is a simple method to find areas where traditional AFP surveillance indicator targets are not reliably met. For areas where AFP surveillance indicator targets are met, systematic assessment of unusual patterns (‘flags’) can be a useful prompt for further investigation and field review.

## 1 Introduction

Acute flaccid paralysis (AFP) surveillance is a critical component of the Global Polio Eradication Initiative (GPEI) [1], [2]. In the presence of a sensitive AFP surveillance system, the absence of detection of polio disease over time indicates absence of poliovirus transmission and informs decisions on certification of the eradication of poliomyelitis [1], [3]. The quality of AFP surveillance affects both the integrity of assessments of progress towards eradication and the grounds for certification [4]. The two principal indicators used to measure the quality of AFP surveillance are the non-polio AFP (NPAFP) rate and stool specimen adequacy [5]. The NPAFP rate, defined as the number of AFP cases aged < 15 years not due to polio per 100,000 children aged < 15 years per year, assesses surveillance system sensitivity to identify cases of paralytic poliomyelitis. While the true rate of non-polio causes of paralysis varies among areas, an NPAFP rate ≥ 2 at the national and first administrative (state/province) subnational level is used as an indicator of sufficient sensitivity in World Health Organization (WHO) regions where wild poliovirus is still endemic [5]. AFP cases are classified as polio or discarded as non-polio primarily based on virologic testing of stool specimens from AFP case-patients and/or their close contacts. Stool specimens are considered adequate for isolation of poliovirus when 1) two stool specimens are collected within 14 days of paralysis onset; 2) stool specimens are collected at least 24 hours apart; and 3) specimens arrive at a WHO-accredited polio laboratory in “good” condition [6]. A specimen is considered to have arrived in “good” condition if of adequate volume, the reverse cold chain has been maintained, and received by a WHO-accredited laboratory without leakage or desiccation [6]. A target of ≥ 80% of AFP cases with adequate specimens is used to assess performance of surveillance sensitivity and specificity [6]. In addition to informing decisions on polio eradication certification, surveillance data are analyzed to identify areas to prioritize resources such as supplemental immunization activities (SIAs), and to estimate effectiveness of interventions [7], [8], [9], [10], [11].

**Fig. 1:**
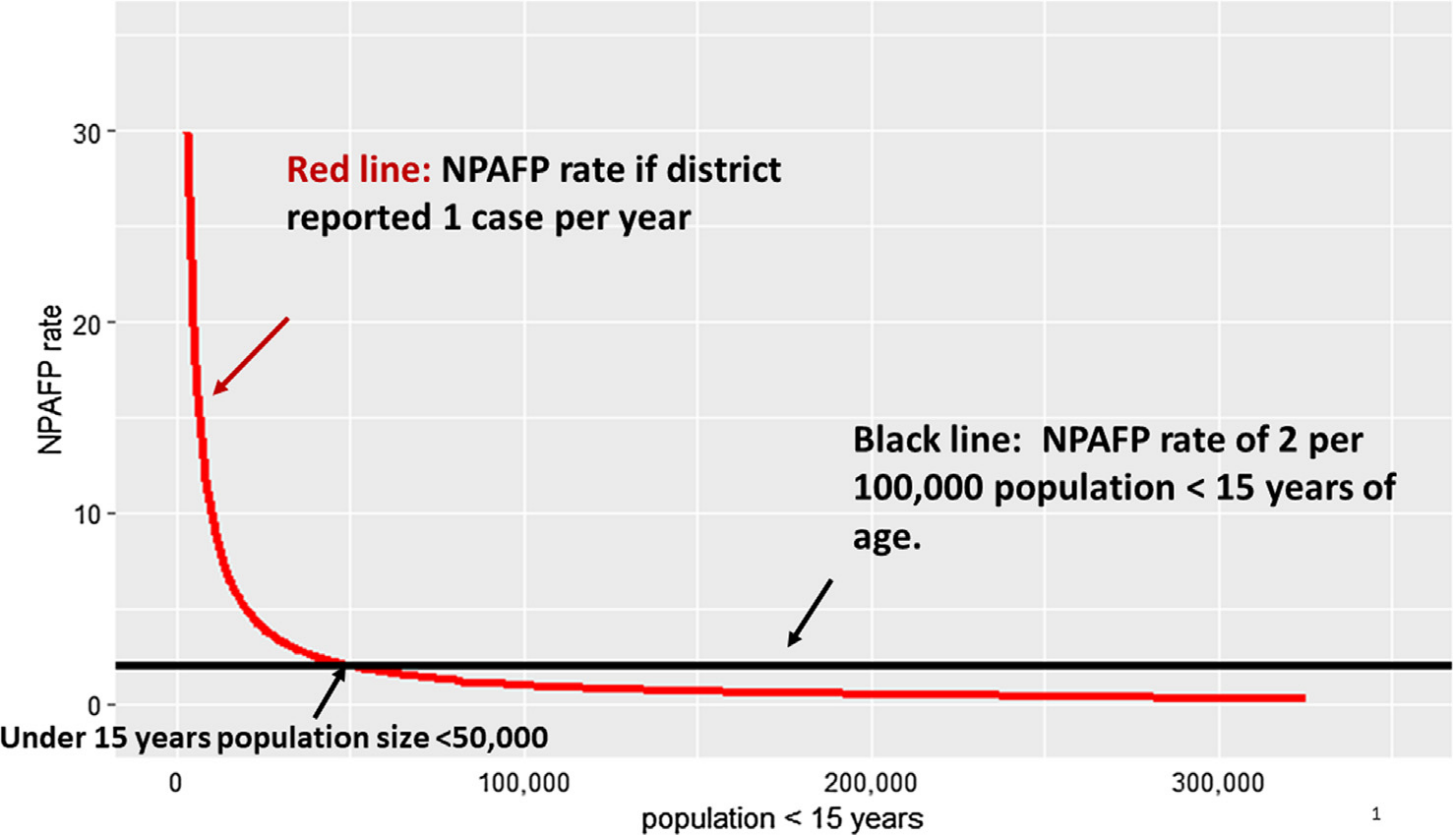
Non-polio Acute Flaccid Paralysis reporting rate for one case reported per year, by population size.

These primary AFP surveillance indicators have some notable limitations. The NPAFP rate indicator is not informative for small populations; on one hand, AFP cases might not occur every year, and so lack of detection might not indicate a poorly functioning system [12]. On the other hand, small numbers of NPAFP cases can also result in nominally high NPAFP rates (Fig. 1). Thus, the number of NPAFP cases in small populations in any 12-month period is difficult to interpret. A second limitation relates to AFP surveillance system processes such as potential for over-reporting of NPAFP (e.g. inclusion of cases that are not true AFP) and data manipulation (e.g. an officer completing the investigation form selectively excludes cases that are detected late). To date, monitoring of AFP surveillance performance has focused on tracking indicators by national and subnational areas and using these reported indicators to detect places with poor surveillance performance [5], [13], [14]. Standardized approaches are not available for use by GPEI to detect performance that is exceptionally good (i.e., potential outliers that are “too good to be true”). Field reviews conducted in Nigeria indicated that some NPAFP cases and specimen collection adequacy based on date of paralysis onset could not be verified [15]. This highlights the need for approaches to identify areas where unexpected patterns in surveillance data (i.e., very high NPAFP rates and very low proportion of AFP cases with collection of stool specimens > 14 days after onset of paralysis, and percentage of missing stool specimens) might indicate a surveillance system that is not functioning as intended.

In their December 2017 report, the Independent Monitoring Board (IMB) of the GPEI stated “… much more emphasis should be given to analysis and verification of the standard indicators of reported AFP rates and stool specimen adequacy, and to closing the gaps in these areas as well as identifying patterns of reporting which are likely to represent data falsification” [16]. In response to both the limitations of available surveillance indicators and to the recommendation of the IMB, we developed two simple new approaches to analyze and present AFP surveillance data which may be used for monitoring and evaluation within the GPEI or within a country’s ministry of health. First, high-level analysis of surveillance indicators (e.g., country- or province-level) can mask gaps in smaller areas, while district-level analyses may be uninformative or misleading when populations are small. To address these limitations, we developed a simple approach for grouping small districts to make uniform, epidemiologically relevant blocks, referred to here as “spatial binning.” Second, even in high quality AFP surveillance systems, the expectation is for some cases to be reported beyond 14 days after onset and some to be missing one or more stool specimen collections. With this in mind, we developed an approach to search for unexpected patterns in surveillance data, termed “surveillance flags analysis.” Surveillance flags point to areas for further investigation; field reviews are essential to understand surveillance processes that lead to unexpected outcomes.

## 2 Methods

### 2.1 Data sources

*S*urveillance data associated with AFP cases came from the WHO Polio Information System (POLIS). POLIS is a case-based standardized data repository of polio surveillance activities across each WHO region of the world, and includes data from 2010-present. POLIS includes information on location (to 2nd administrative unit), paralysis onset, notification and investigation dates, stool collection dates, lab results, as well as basic clinical features found during the investigation and follow-up, if conducted. POLIS was the source for AFP cases, national and first administrative level (state/province) population estimates and geographic boundaries used for these analyses. We limited our analysis to endemic and recently endemic WHO regions (African, Eastern Mediterranean, and South East Asian Regional Offices). To supplement these population estimates from POLIS, second administrative level (district) population data came from WorldPop [17], an open-access spatial collection that provides demographic datasets. All descriptive analyses were conducted using R version 3.3 [18].

### 2.2 Spatial binning

Our goal was to evaluate AFP surveillance performance indicators in subnational areas that would be comparable across countries. In order to do this, we created relatively uniform areas of 200,000 individuals under 15 years of age. The figure 200,000 was arbitrarily chosen because it is large enough so that multiple NPAFP cases would be expected in an area in a given year, and small enough so that epidemiologically relevant populations are less likely to be masked. To do this, we started with the smallest available administrative unit (districts), and iteratively: 1) selected the area with the smallest population; 2) merged that area with a contiguous area in the same country with the next smallest under-15 population and 3) repeated until all merged areas had >200,000 individuals under 15 years of age. The resulting merged areas were used as the basis for analysis, referred to as aggregated districts or spatial bins. The unit of analysis will affect results and interpretation, and other binning approaches are possible, for instance, merging districts which are closest to each other, which we explore in the Supplementary Material. Particularly if used at the country level, additional criteria may be desirable such as administrative or social factors. We summarized surveillance performance by calculating the proportion of areas meeting both NPAFP rate and stool specimen adequacy indicators. For comparison, we also summarized surveillance proportion of provinces (first administrative level) and districts (second administrative level) meeting both stool specimen adequacy and NPAFP rate indicators.

### 2.3 Surveillance flags analysis

We developed surveillance indicators of concern or “flags” by conducting descriptive analyses of data from 49 countries included in POLIS with >250 cumulative AFP cases reported during a 3-year period (2015 – 2017), chosen to provide stability in estimates. For each country, we examined the distribution of cases from the onset of paralysis to collection of second stool specimen, the distribution of cases from onset to notification, the percentage of cases missing any stool specimen collection, and the distribution of cases by age in years. In each instance, we compared the distributions across all countries included in the analysis, and selected prioritization thresholds for each distribution. These thresholds were based on: 1) the distribution patterns for the 49 countries; and 2) the expectation that even the strongest surveillance system cannot function perfectly all the time (e.g., delayed case notification and missed stool specimen collection). In short, these arbitrary thresholds were chosen to identify unexpected patterns in AFP surveillance data, with the goal of identifying areas for further follow-up and/or investigation. To aid in interpretation of each surveillance flag, we developed standardized data visualizations to show the distribution of cases underlying each flag.

### 2.4 Timeliness flag

Given the challenges in identification of cases and timely investigation our expectation was that collection of two stools within 14 days of onset of paralysis is not always possible. To detect countries with unexpected patterns, we flagged countries where ≤ 3.0% of AFP cases had two stools collected after 14 days.

### 2.5 Late notification flag

We assumed that some cases would be reported very late (notification > 60 days after onset; e.g., late detection based on health facility record review). We flagged countries with < 1 in 30 cases notified > 60 days after onset, among cases notified > 14 days after onset. This corresponds to a proportion of ≤ 0.03 (or ≤ 3%) (cases with onset to notification > 60 days / cases with onset to notification > 14 days).

### 2.6 Missing stool specimen flag

The global standard practice for cases identified > 14 days after onset is to investigate all AFP cases identified < 6 months after onset, and to collect stool specimens in those with onset within 60 days. It may be difficult to collect any stool specimens for all AFP cases. We flagged countries with ≤ 0.003 (or ≤ 0.3%) of cases missing any specimen among cases ≤ 60 days from onset to notification.

### 2.7 Age flag

The case definition of AFP and the target NPAFP rate require detection of all cases in children <15 years of age. Globally, in addition to routine reporting of AFP cases from health facilities, cases are often detected during SIAs. These cases tend to be among children under age 5 years as SIAs are generally targeted to that age group. We flagged countries with < 1 in 5 NPAFP cases among children > 5 years old. This corresponds to a ratio of ≥ 4.0 (age in years at onset of paralysis < 5 years: 5–14 years).

### 2.8 Combining spatial binning and surveillance flags analysis

In a final step, we combined the subnational analysis and surveillance flag analysis approaches to assess AFP surveillance performance for 2017 for the 49 countries assessed for surveillance flags in the WHO African (AFR), Eastern Mediterranean (EMR) and South-East Asia (SEAR) regions, and compared this to a traditional approach to assessing surveillance performance based on NPAFP indicators alone.

## 3 Results

### 3.1 Spatial binning

Among the 79 countries and areas in AFR, EMR, and SEAR analyzed, there were 8,725 districts, with a median population of 60,140 individuals under 15 (IQR 23,550 – 134,500). Applying our method resulted in 2,712 aggregated districts with a median population of 376,700 individuals under the age of 15 (IQR 273,200 – 547,100). By comparison, 1,489 provinces had a median population of 363,500 under age 15 years (IQR 126,300 – 861,700 thousand). Thus, the aggregated districts tended to be comparable in size to provinces but less variable.

**Fig. 2:**
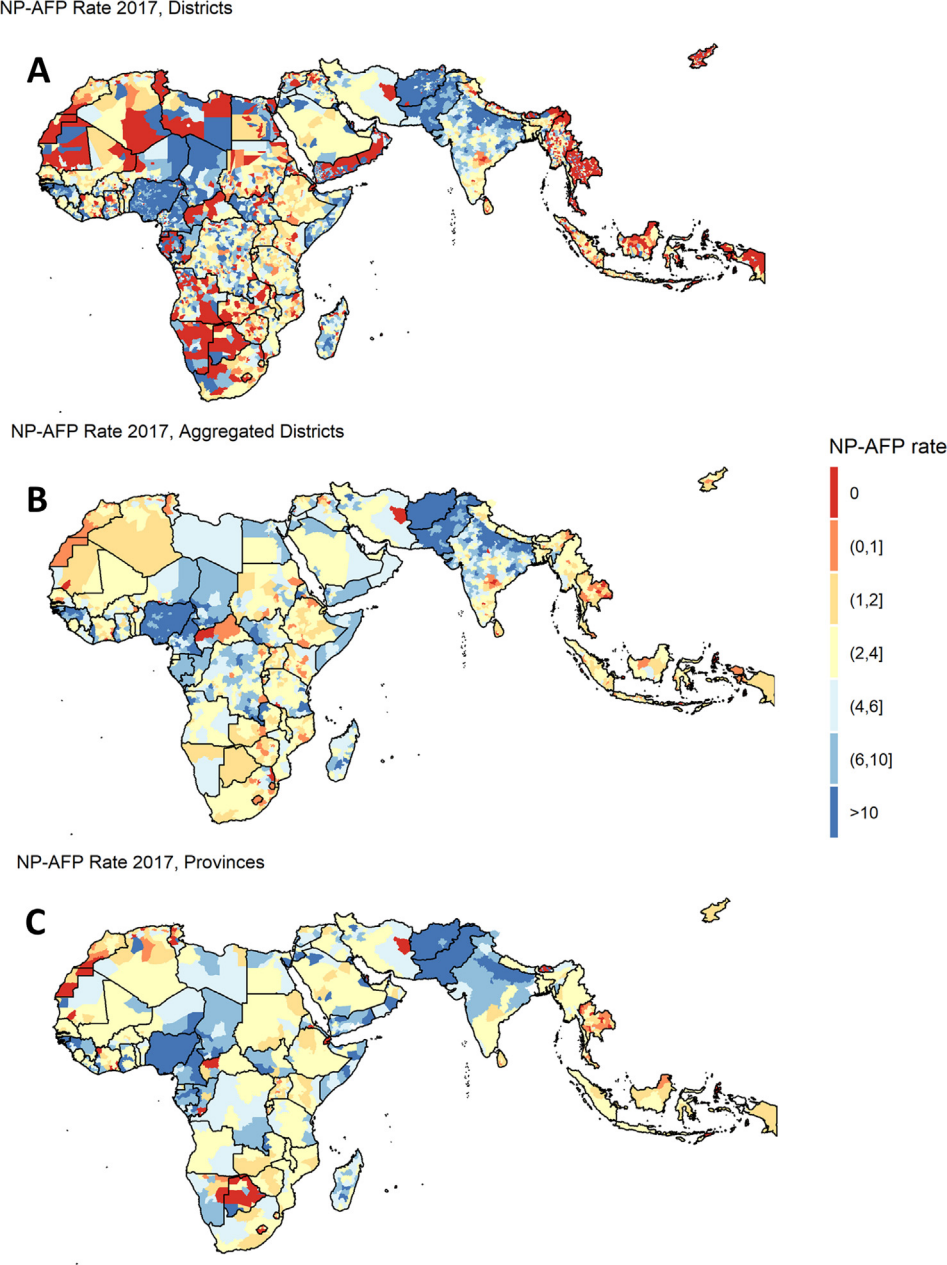
NPAFP Rates in Subnational Areas.

Fig. 2 illustrates the NPAFP rates for 2017 using different units of analysis: A. districts, B. spatial binning, and C. provinces. When using districts as the unit of analyses, many countries have geographically large districts which did not report any NPAFP cases during the year. However, many of these districts have small populations, and upon aggregation do not show evidence of gaps. Of note, analyzing data at the province level in large countries can obscure meaningful differences at the sub-national level. For instance, no provinces in the Democratic Republic of the Congo (DRC) have NPAFP rates below 2, while there are aggregated districts which have substantially lower rates.

**Fig. 3:**
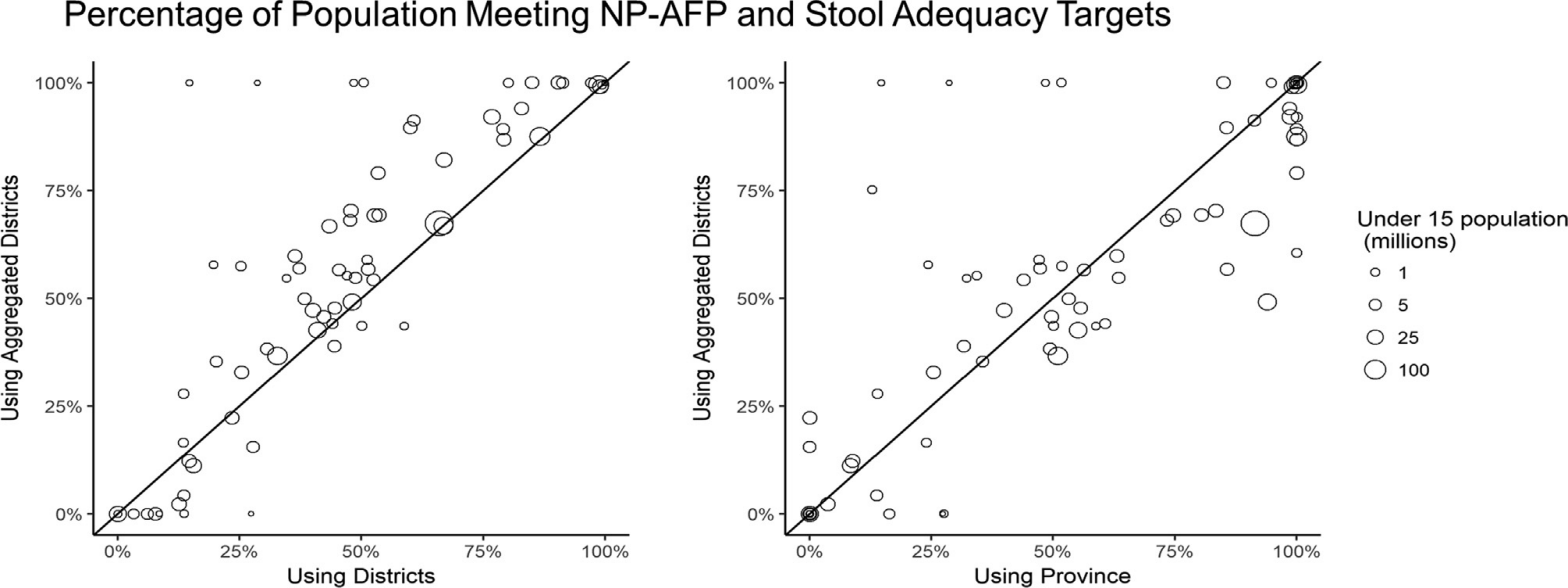
Percentage of the Population in Each Country Meeting Non-Polio Acute Flaccid Paralysis Reporting Rates and Stool Adequacy Targets.

Differences in subnational surveillance indicators can become important when comparing countries for evaluation and prioritization of interventions. Fig. 3 shows the percentage of the population meeting both NPAFP and stool adequacy targets, using different units of analysis. Relative to a district-level analysis, spatial binning usually results in a higher percentage of the population meeting both indicators, particularly in small countries. For instance, in the Republic of the Congo, only 25% of the population lived in districts where both surveillance targets were met, while when using spatial binning 57% of the population lived areas where both surveillance targets were met. There, using districts as the unit of analysis overstates surveillance gaps, since the median district in our analyses has only 42 thousand children under 15 years of age. On the other hand, analyses relying on province level data tend to mask gaps at subnational levels, particularly in large countries with large provinces (e.g., DRC, India, and Nigeria). Overall, 31/79 countries had > 80% of the population living in areas meeting both indicators when using spatial binning, compared with only 15/79 when using districts as the unit of analysis, or 35/79 when using provinces as the unit of analysis. Details on the number of AFP cases (2017), under 15 population, national NPAFP rate and stool adequacy, and the percentage of population living in areas meeting NPAFP and stool adequacy indicators at the province-, district-, and aggregated subnational-group are provided in the Supplementary Material.

### 3.2 Surveillance flags

We analyzed AFP surveillance data from 79 countries and areas in AFR, EMR, and SEAR, including in the final analysis 49 countries with ≥ 250 AFP cases reported from 2015 to 2017^1^. Of the 49 countries, the median number of reported AFP cases from 2015 to 2017 was 1229, with a range of 299 (Lebanon) to 132,917 (India). *Timeliness:* The median percentage of cases with > 14 days from paralysis onset to second stool collection was 8.4%, with a range of 0 (Democratic People’s Republic of Korea) to 24.5% (Thailand) (see the Supplementary Material). Overal, nine countries (18%) were flagged for timeliness by reporting ≤ 3% of AFP > 14 days from onset to second stool specimen. *Late notification*: The median proportion of cases with onset to notification > 60 days relative to cases with onset to notification > 14 days was 0.09, with a range of 0 (Central African Republic, Iran, Jordan, Mali, and Saudi Arabia)^2^ to 0.36 (Rwanda). Overall, twelve countries (24%) were flagged with a proportion ≤ 0.03. *Missing stool specimens*: The median percent of cases missing any stool specimen among cases ≤ 60 days from onset to notification was 0.4%, with a range of 0 (Benin, Burkina Faso, Democratic People’s Republic of Korea, Jordan, Myanmar, Rwanda, Senegal, Togo, United Republic of Tanzania, Zambia) to 10.5%, (South Africa). Twenty countries (41%) were flagged for reporting ≤ 0.3% of cases missing any stool specimen. *Age*: The median ratio of cases with age in years at onset of paralysis < 5: age in years at onset of paralysis 5–14 years was 1.8, with a range of 0.6 (Myanmar) to 7.1 (Somalia). Four countries (8%) had a ratio of ≥ 4.0, and were flagged for age distribution. In total, 20 countries were not flagged, 18 countries had one flag, 6 countries had two flags, and 5 countries had three surveillance flags.

**Fig. 4:**
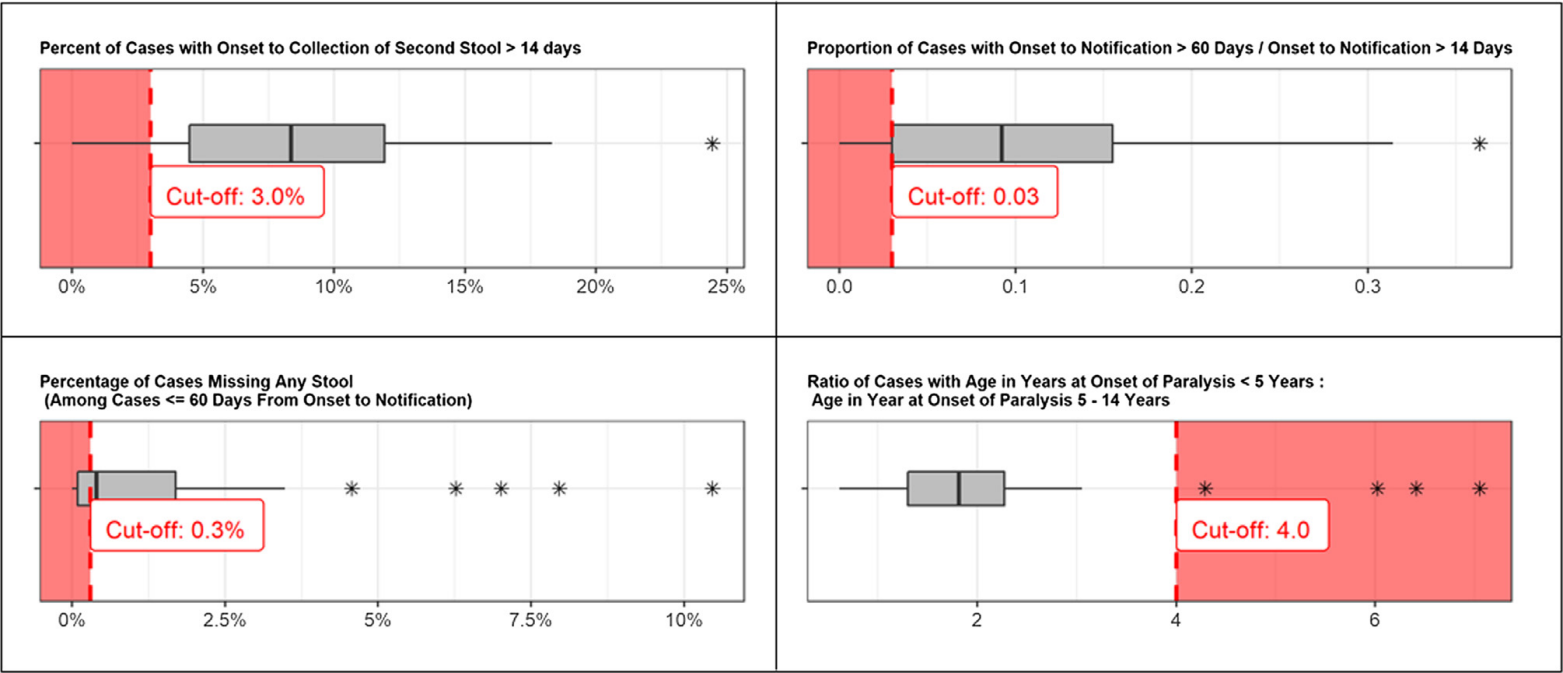
Distribution of Indicators by Type of Surveillance Flag, 49 countries in the African, Eastern Mediterranean, and South East Asian regions with ≥ 250 AFP cases reported from 2015 to 2017.

**Fig. 5:**
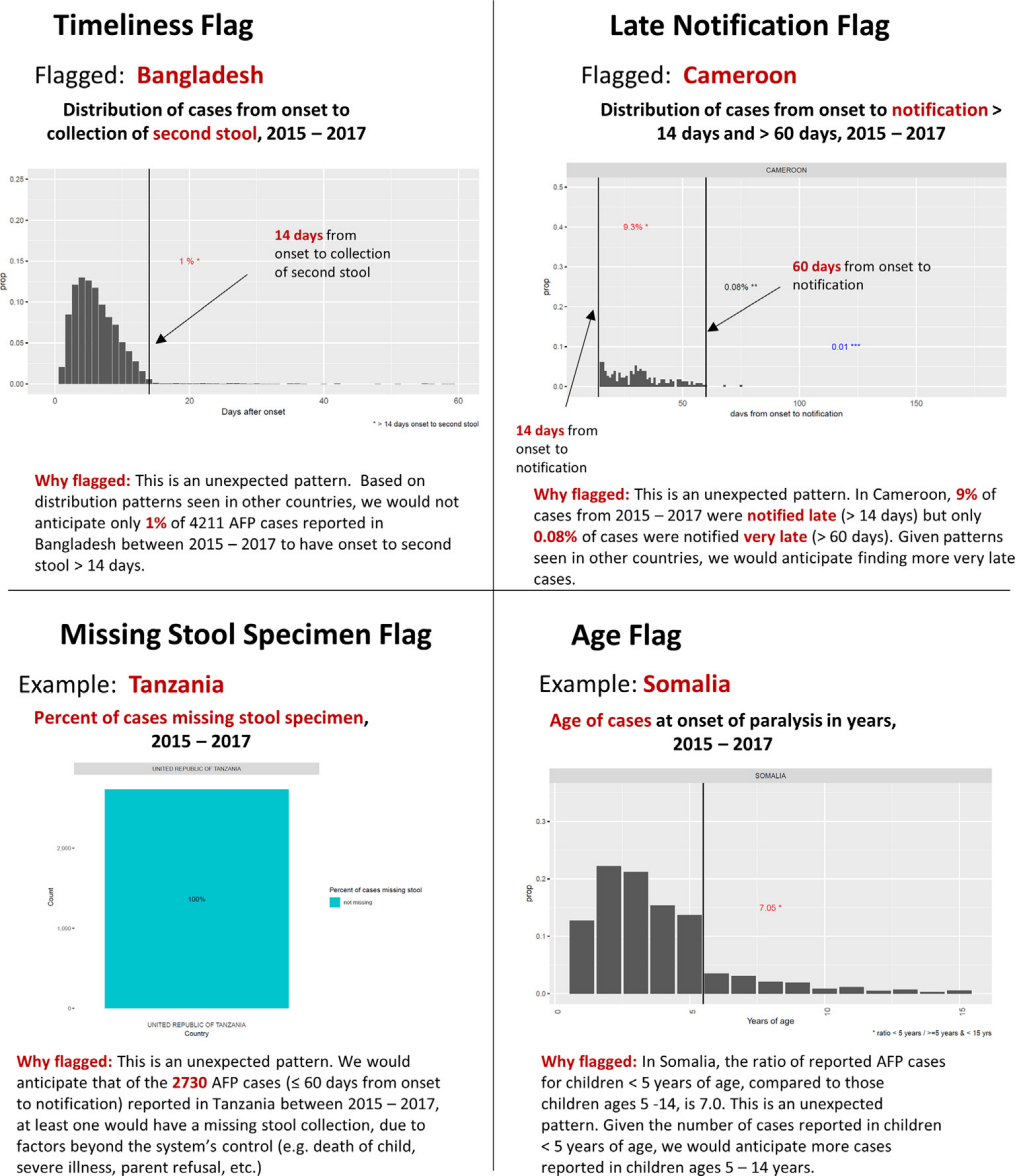
Example of Standardized Data visualizations for Four Surveillance Flags, 2015 – 2017.

Distributions of indicators by type of surveillance flags for each of the 49 countries included in the final analysis are presented in Fig. 4, and distributions by region for all 79 countries in AFR, EMR and SEAR is included in Figure A2 of the Supplementary Material. Additional data on the number of AFP cases from 2015 to 2017, the percentage of cases missing any stool collection, and distribution of cases by onset to notification, onset to second stool, and by age at onset of paralysis for all 79 countries in AFR, EMR, and SEAR is included in Table A2 of the Supplementary Material. Fig. 5 provides an example of standardized data visualizations and explanatory text for each of the surveillance flags.

### 3.3 Combining spatial binning and surveillance flags

**Fig. 6:**
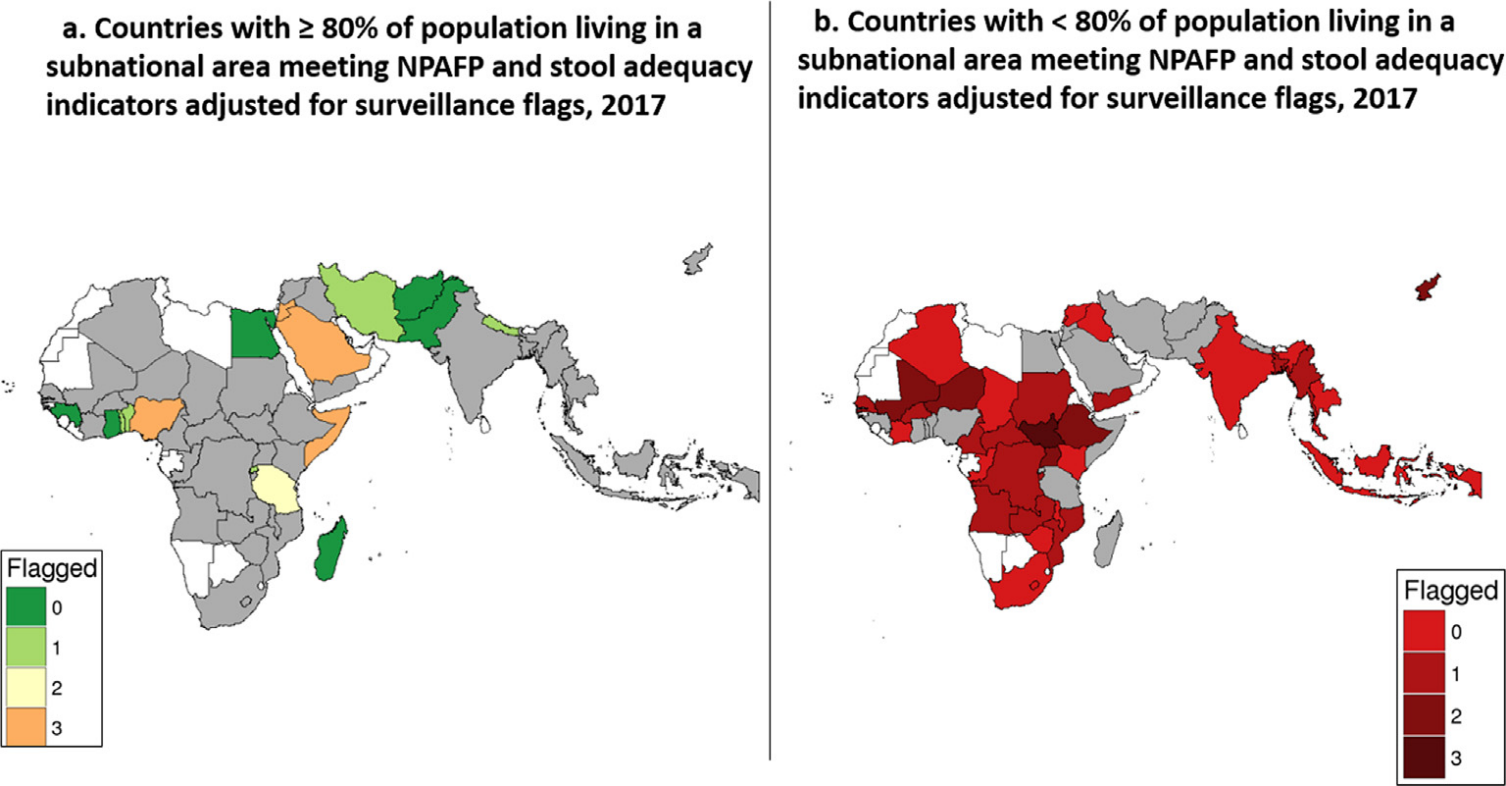
Countries Living in a Subnational Area Meeting NPAFP and Stool Adequacy Indicators Adjusted for Surveillance Flags, 2017.

Fig. 6 is a map of the 49 countries meeting the inclusion criteria for the spatial binning and surveillance flags. We compare a traditional approach to displaying AFP surveillance indicators by combining spatial binning and surveillance flags. There are 16 countries with ≥ 80% of population living in provinces meeting NPAFP rate and stool specimen adequacy indicators, shown in Fig. 6a. Of these 16 countries with reported good performance, six countries (38%) have zero surveillance flags; six countries (38%) have one or two surveillance flags; and four countries (25%) have three surveillance flags. Another 33 countries with < 80% of population living in provinces meeting NPAFP rate and stool specimen adequacy indicators are shown in Fig. 6b. Among these countries with reported poor performance, 14 countries (42%) have zero surveillance flags; 18 countries (55%) have one or two surveillance flags; and 1 country (3%) had three surveillance flags.

## 4 Discussion

Analysis of standard AFP surveillance indicators play a critical role in assessing polio surveillance, but there are notable limitations to their use and interpretation. When assessed at the national level, a country’s failure to meet one or both indicators can highlight a high-level need for surveillance strengthening. However, national-level indicators alone are not sufficient to determine surveillance system quality, and so appropriate analysis is subnational, generally directed to province-level performance indicators. Careful spatial binning and flagging unusual data offer additional ways to better understand and assess surveillance system performance and should be part of any thorough analysis of surveillance performance. While these methods may be applied independently, there are advantages to combining these approaches to strengthen the standard analysis of surveillance performance indicators. In the analysis of public health surveillance data, certain types of errors may be associated with data manipulation, however, an assessment of the quality of data needed for appropriate inferences is often lacking [19], [20]. In comparison to standard approaches, our approaches provide a more nuanced view of surveillance performance, and highlight a need for closer examination of surveillance system processes. Additionally, these methods, which may be used by researchers globally or by program staff in WHO regional and country offices, or other GPEI partners, will be most effective when applied in conjunction with field reviews. For example, a small field review in Nigeria found that, based on a reassessed date of onset, some reportedly adequate stool specimens were in fact inadequate stool specimens, which has been substantiated by subsequent reviews by the country team [15]. These approaches may be particularly useful in prompting further investigation in situations where a country meets surveillance indicators when assessed by traditional methods, but is highlighted by these other methods.

Analyzing spatially binned data is a simple method which allows for a more reliable representation of surveillance performance, particularly for low population districts. For example, when using districts as the unit of analyses, many countries have districts with small populations which did not report any NPAFP cases during the year. Using the spatial binning, these areas did not show evidence of gaps.

Surveillance flags were found in both countries with poor surveillance performance (< 80% of the population living in subnational areas meeting both indicators) and countries with good surveillance performance (≥ 80% of the population living in a subnational area meeting both indicators). In this analysis, we applied surveillance flags at the national level, but this approach can also be applied at the province level, if the reported number of AFP cases during a given time period allows for the detection of unexpected patterns. The presence or absence of surveillance flags alone does not indicate a poor- or well-performing surveillance system. Instead, surveillance flags are best interpreted in conjunction with performance indicators. In addition, the results of surveillance flags analysis should be interpreted with caution – surveillance flags point to areas for further investigation of surveillance activities to elucidate reasons for observed unexpected patterns. Importantly, unexpected performance may indeed represent extremely well-functioning surveillance systems (positive outliers) that might be examples of high performance, providing lessons learned that could be shared with other countries. Importantly, even if subnational indicators are met and there are no flags raised, high quality surveillance cannot be assured apart from proper field supervision and quality oversight. Surveillance flags help to identify areas for further investigation, but adequate supervision and true, thorough active surveillance visits are required everywhere, not only where flags are raised.

There are limitations to the methods used for the new AFP surveillance indicator analysis approaches. Specifically, while spatial binning improves the reliability of the standard indicators, it doesn’t address the basic concerns regarding AFP surveillance indicators: it remains difficult to assess performance in small populations, and reporting an expected number of NPAFP cases does not assure that all true AFP were detected. For instance, a system might meet the indicators without covering the entire population, may meet the indicators by only including cases that were properly investigated, or a surveillance officer may stop conducting active surveillance health facility visits once a “quota” is met. An alternative to our approach of spatial binning would be to use spatial and temporal smoothing [21]. While smoothing methods can be statistically preferable, they require specialized software to implement, and because information is shared between neighboring areas the evaluation cannot be attributed to administrative units and those responsible for surveillance in them. Additionally, spatial smoothing would introduce bias in important non-smooth scenarios, such as when areas are inaccessible because of insecurity. Smoothing over time would also allow evaluation of small areas, but would require incorporation of distant time points which may be less relevant to current performance. For evaluation of very small areas, process indicators, such as active case search, may be preferable. Our method of merging districts was based on population and neighboring districts; an alternative method would be to group areas according to shared characteristics by experts familiar with the area. Likewise, surveillance flags can only detect unexpected patterns – but they cannot provide insight into why these patterns occur. Additionally, our surveillance flags were applied to areas where the number of AFP cases (> 250) allows for the potential detection of unexpected patterns. Thus, these methods are limited to areas with larger populations, or smaller areas over a longer period of time. Also, surveillance flags only capture longer-term patterns, and cannot provide a measure of short-term change or improvements. Finally, the thresholds for our surveillance flags were not determined by statistical methods; instead, cutoffs were chosen based on both observed patterns (e.g., country-level distributions) and programmatic needs (e.g., to identify areas with potential surveillance abnormalities). In the future, as evidence from field reviews accumulates, these thresholds might be adjusted accordingly

## Declaration of Competing Interest

The authors declare that they have no known competing financial interests or personal relationships that could have appeared to influence the work reported in this paper.

## Footnotes

1 51 countries met the inclusion criteria of ≥ 250 AFP cases reported from 2015 to 2017. In two countries (Eritrea and Morocco), however, the number of AFP cases fell below 250 when running flag specific analysis (e.g., <250 cases ≤ 60 days from onset to notification). These countries were not included in the results.

2 The Democratic People’s Republic of Korea did not report any cases with onset to notification > 14 days (the denominator for this calculation) from 2015 to 2017, and was not flagged.
