# Supplementary material for: New analytic approaches for analyzing and presenting polio surveillance data to supplement standard performance indicators
Source: Vaccine X. 2020 Mar 21;4:100059. doi: 10.1016/j.jvacx.2020.100059 (PMC7090369; doi:10.1016/j.jvacx.2020.100059)
Supplement: Supplementary data 1 [file mmc1.docx]

**Appendix**

**Comparison between different aggregation methods**


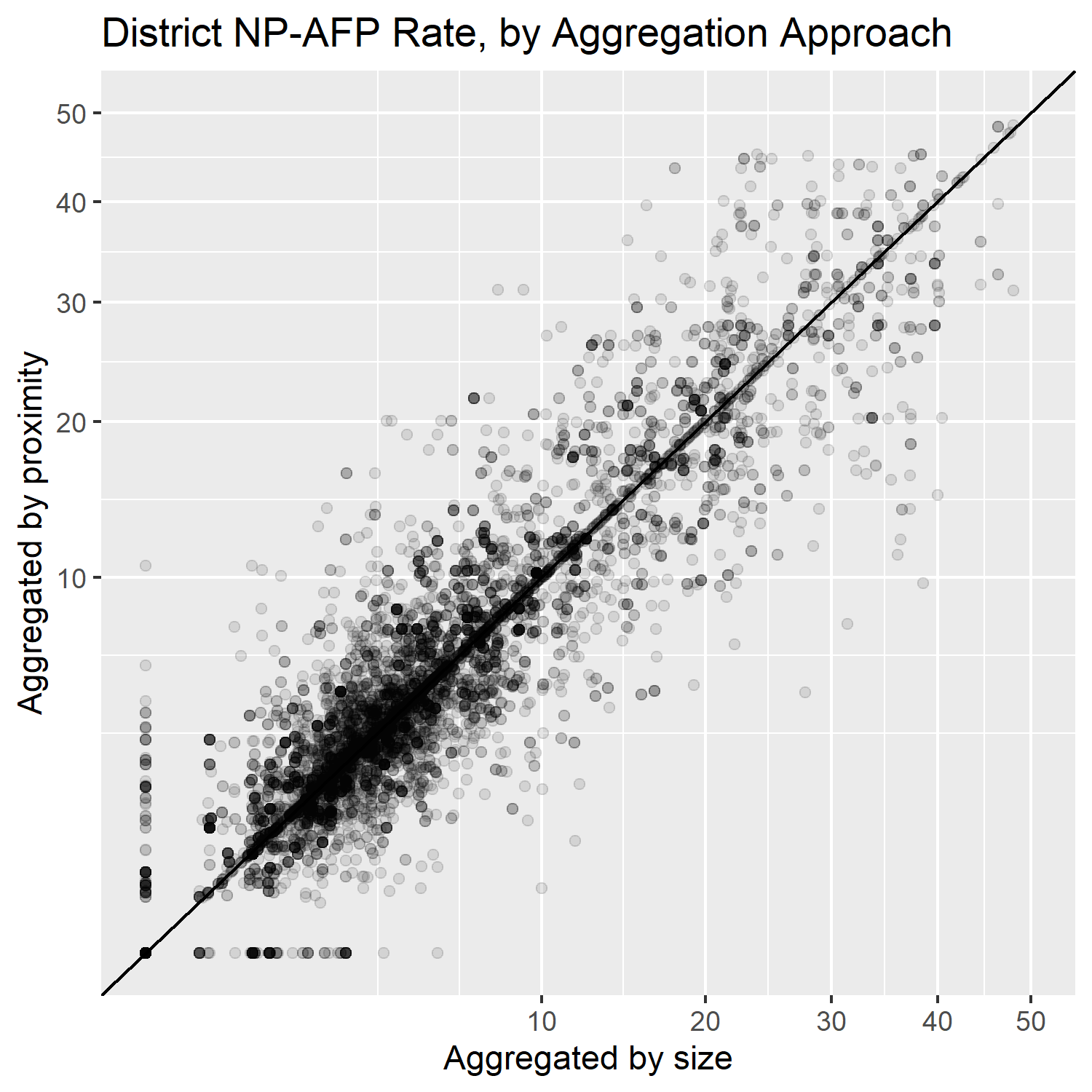

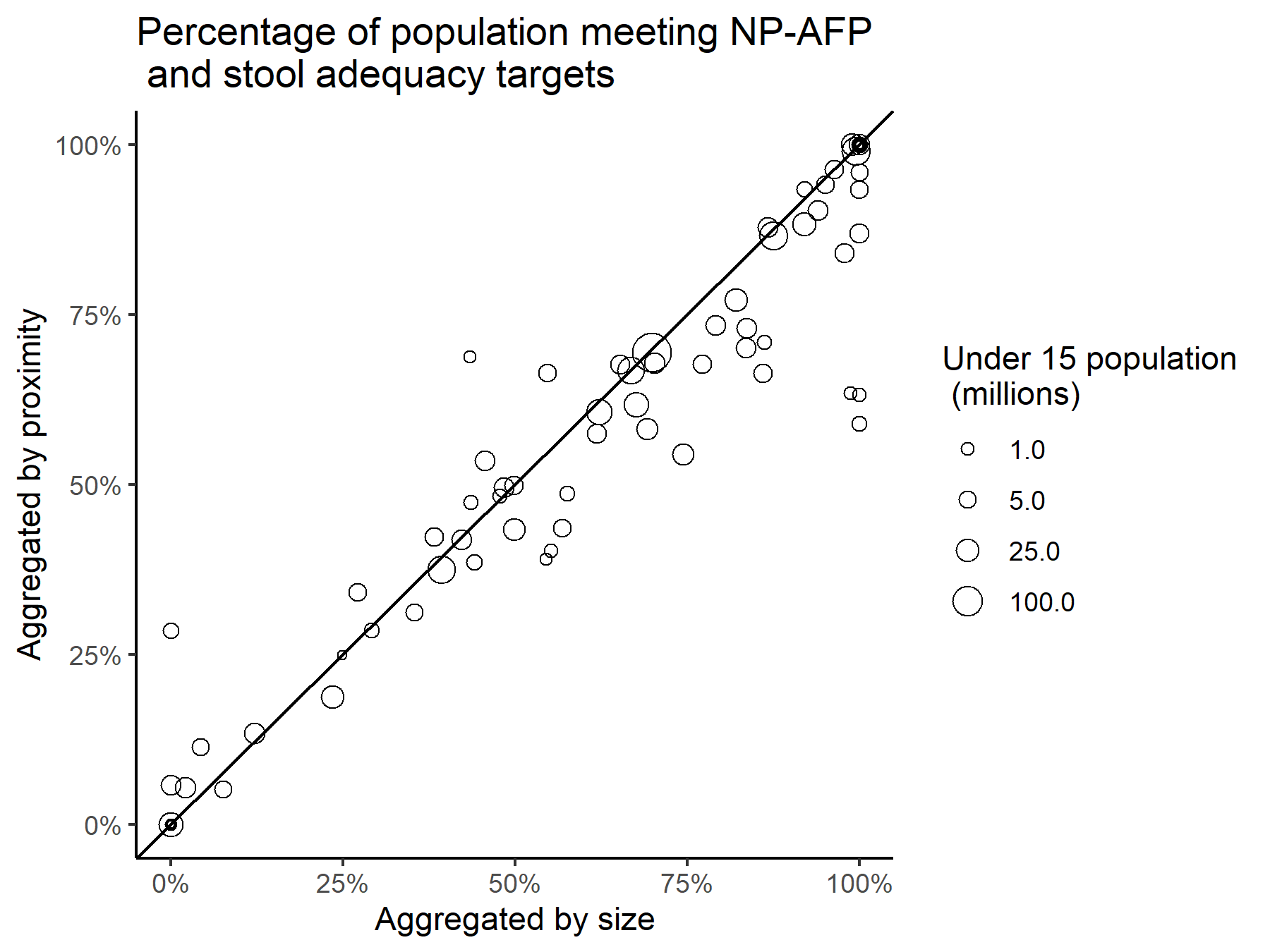
As suggested by a reviewer, we examined the impact of different aggregation methods. In the manuscript we joined the smallest district with its smallest adjacent district until all had > 200k under-15. Here we try an alternate aggregation approach by (1) selecting the smallest district (2) merge it with the closest adjacent district, by centroid distance (3) re-calculate the centroid for the merged districts, and (4) repeat until all districts have > 200k individuals under-15. We find that the approaches produce similar NP-AFP rates for individual areas (R-squared = 0.93), as well as similar proportions of the population meeting both indicators (R-squared = 0.9).

**Figure A1:** Comparison between different aggregation methods.

**Table A1:** Population living in areas meeting NPAFP and Stool Adequacy Indicators in 2017 with Province, District and Sub-National grouping.

|  |  |  |  |  | **Population living in areas meeting NPAFP and stool adequacy indicators (%)** | | |
| --- | --- | --- | --- | --- | --- | --- | --- |
| **Country** | **No. of AFP cases (2017)** | **National NPAFP rate** | **Stool Adequacy (%)** | **Under 15 Population** | **province-level** | **district-level** | **spatial binning** |
| AFGHANISTAN | 3,080 | 20.3 | 93.5 | 15,136,792 | 100% | 90% | 100% |
| ALGERIA | 287 | 2.6 | 78 | 10,972,157 | 25% | 25% | 33% |
| ANGOLA | 409 | 3.4 | 96.9 | 11,891,167 | 100% | 53% | 79% |
| BAHRAIN | 18 | 6.7 | 100 | 266,807 | 100% | 100% | 100% |
| BANGLADESH | 1,361 | 2.4 | 98.7 | 56,199,168 | 91% | 67% | 67% |
| BENIN | 213 | 3.9 | 90.1 | 5,435,260 | 91% | 61% | 91% |
| BHUTAN | 10 | 3.7 | 81.8 | 268,588 | 29% | 29% | 100% |
| BOTSWANA | 14 | 1.8 | 92.9 | 760,587 | 32% | 35% | 55% |
| BURKINA FASO | 308 | 3.7 | 85.1 | 8,387,072 | 73% | 48% | 68% |
| BURUNDI | 141 | 2.6 | 84.1 | 5,374,044 | 35% | 20% | 35% |
| CAMEROON | 969 | 9.3 | 85.5 | 10,372,344 | 80% | 54% | 69% |
| CENTRAL AFRICAN REPUBLIC | 164 | 7.4 | 80.2 | 2,207,582 | 47% | 51% | 59% |
| CHAD | 532 | 7.6 | 78.8 | 7,041,626 | 44% | 52% | 54% |
| CIV | 313 | 3.3 | 87.9 | 9,600,795 | 56% | 45% | 48% |
| COMOROS | 34 | 11.8 | 94.1 | 287,872 | 100% | 100% | 100% |
| CONGO | 111 | 5.5 | 83.8 | 2,002,600 | 52% | 25% | 57% |
| DEMOCRATIC PEOPLE'S REPUBLIC OF KOREA | 90 | 1.5 | 98 | 5,880,701 | 0% | 28% | 15% |
| DEMOCRATIC REPUBLIC OF THE CONGO | 2,048 | 5.2 | 78.7 | 39,430,423 | 55% | 41% | 43% |
| EGYPT | 1,219 | 4.3 | 94.4 | 28,524,322 | 99% | 77% | 92% |
| EQUATORIAL GUINEA | 10 | 3.3 | 16.7 | 303,141 | 0% | 9% | 0% |
| ERITREA | 102 | 4.7 | 97.1 | 2,175,763 | 75% | 43% | 66% |
| ETHIOPIA | 1,070 | 2.3 | 86.1 | 45,720,242 | 94% | 48% | 49% |
| GABON | 51 | 7.6 | 56.9 | 669,655 | 28% | 14% | 0% |
| GAMBIA | 18 | 2.1 | 83.3 | 876,175 | 13% | 8% | 75% |
| GHANA | 522 | 5.2 | 88.1 | 10,021,453 | 86% | 60% | 90% |
| GUINEA | 452 | 7.5 | 87.6 | 6,053,625 | 100% | 79% | 89% |
| GUINEA-BISSAU | 85 | 10.5 | 81.2 | 809,866 | 59% | 59% | 43% |
| INDIA | 38,312 | 9.7 | 85.5 | 396,649,281 | 91% | 66% | 67% |
| INDONESIA | 1,612 | 2.3 | 81.7 | 69,221,813 | 51% | 33% | 37% |
| IRAN (ISLAMIC REPUBLIC OF) | 820 | 4 | 96.5 | 20,732,292 | 99% | 99% | 99% |
| IRAQ | 657 | 4.1 | 86.8 | 16,031,699 | 75% | 53% | 69% |
| JORDAN | 115 | 4.3 | 100 | 2,698,653 | 100% | 97% | 100% |
| KENYA | 435 | 2.3 | 82.6 | 19,282,949 | 53% | 36% | 43% |
| KUWAIT | 69 | 9.2 | 91.3 | 746,437 | 99% | 99% | 99% |
| LEBANON | 75 | 5.6 | 80 | 1,341,804 | 34% | 47% | 55% |
| LESOTHO | 8 | 1.1 | 100 | 716,081 | 30% | 30% | 0% |
| LIBERIA | 81 | 4.3 | 81.5 | 1,865,047 | 50% | 50% | 44% |
| LIBYA | 86 | 4.6 | 96.5 | 1,876,087 | 95% | 80% | 100% |
| MADAGASCAR | 703 | 6.1 | 92.7 | 11,492,713 | 98% | 83% | 94% |
| MALAWI | 287 | 3.7 | 79.9 | 7,845,001 | 56% | 45% | 57% |
| MALI | 253 | 2.8 | 86.2 | 9,177,089 | 86% | 51% | 57% |
| MAURITANIA | 49 | 2.6 | 73.5 | 1,866,744 | 24% | 13% | 16% |
| MAURITIUS | 8 | 3.3 | 62.5 | 243,503 | 27% | 27% | 0% |
| MOROCCO | 142 | 1.3 | 57 | 10,931,266 | 0% | 8% | 0% |
| MOZAMBIQUE | 356 | 2.9 | 82.8 | 12,255,665 | 50% | 42% | 46% |
| MYANMAR | 391 | 2.8 | 94.9 | 13,895,685 | 83% | 48% | 70% |
| NAMIBIA | 24 | 2.8 | 74.2 | 854,912 | 24% | 20% | 58% |
| NEPAL | 369 | 3.4 | 98.1 | 10,781,803 | 100% | 79% | 87% |
| NIGER | 477 | 4.3 | 72.9 | 11,217,382 | 0% | 23% | 22% |
| NIGERIA | 16,453 | 21.7 | 98.1 | 75,951,006 | 100% | 99% | 100% |
| OMAN | 40 | 4.4 | 90 | 918,570 | 70% | 52% | 99% |
| PAKISTAN | 10,269 | 12.6 | 85.6 | 81,432,605 | 100% | 87% | 88% |
| QATAR | 8 | 2.4 | 100 | 338,816 | 15% | 15% | 100% |
| RWANDA | 137 | 2.7 | 95.6 | 5,047,332 | 100% | 59% | 82% |
| SAUDI ARABIA | 276 | 3.1 | 98.9 | 8,906,355 | 85% | 85% | 100% |
| SENEGAL | 151 | 2.4 | 85.4 | 6,388,044 | 47% | 37% | 57% |
| SIERRA LEONE | 73 | 2.7 | 75.3 | 2,679,570 | 61% | 44% | 44% |
| SOMALIA | 344 | 6.4 | 99.1 | 5,351,273 | 100% | 91% | 100% |
| SOUTH AFRICA | 458 | 3.1 | 66.3 | 15,007,497 | 9% | 15% | 12% |
| SOUTH SUDAN | 384 | 6.5 | 85.6 | 5,904,202 | 63% | 49% | 55% |
| SRI LANKA | 69 | 1.4 | 84.3 | 5,101,819 | 14% | 14% | 4% |
| SUDAN | 521 | 3 | 96.5 | 17,249,256 | 77% | 43% | 67% |
| SWAZILAND | 14 | 2.3 | 100 | 610,412 | 48% | 48% | 100% |
| SYRIAN ARAB REPUBLIC | 285 | 4.2 | 70.1 | 6,806,005 | 32% | 44% | 39% |
| THAILAND | 166 | 1.2 | 68.7 | 13,753,810 | 4% | 13% | 2% |
| TIMOR-LESTE | 1 | 0.2 | 33.3 | 508,468 | 0% | 0% | 0% |
| TOGO | 118 | 3.5 | 97.5 | 3,336,561 | 100% | 72% | 92% |
| TUNISIA | 31 | 1.1 | 80.6 | 2,873,356 | 16% | 3% | 0% |
| UGANDA | 599 | 3 | 85.9 | 20,274,284 | 40% | 40% | 47% |
| UNITED ARAB EMIRATES | 58 | 4.8 | 89.7 | 1,206,516 | 52% | 50% | 100% |
| UNITED REPUBLIC OF TANZANIA | 866 | 3.9 | 97 | 22,475,373 | 97% | 67% | 82% |
| WEST BANK AND GAZA STRIP | 40 | 2.1 | 97.5 | 1,937,192 | 100% | 42% | 61% |
| YEMEN | 605 | 5.6 | 81.9 | 10,767,699 | 63% | 36% | 60% |
| ZAMBIA | 239 | 3.2 | 86.7 | 7,553,957 | 49% | 31% | 38% |
| ZIMBABWE | 173 | 2.3 | 88.3 | 7,412,677 | 53% | 38% | 50% |

**Table A2:** Expanded view surveillance flags by country^[[1]](#footnote-1)^ from 2015-2017.

| **Country** | **No. AFP Cases (2015 - 2017)** | **Timeliness** | **Late Notification** | **Missing Stool** | **Age Flag** | **Total Number of Flags** |
| --- | --- | --- | --- | --- | --- | --- |
|  |  | % cases > 14 days onset to second stool | Proportion of cases with onset to notification > 60 days / onset to notification > 14 days | % of cases missing any stool (among cases <= 60 days from onset to notification) | Ratio of cases with age in years at onset of paralysis < 5 years : age in year at onset of paralysis 5 -14 years) |  |
| AFGHANISTAN | 8,737 | 5.05 | 0.07 | 1.86 | 2.11 | 0 |
| ALGERIA | 1,460 | 12.01 | 0.1 | 4.57 | 1.78 | 0 |
| ANGOLA | 1,228 | 4.49 | 0.05 | 0.08* | 2 | 1 |
| BANGLADESH | 4,211 | 1* | 0.13 | 0.57 | 1.47 | 1 |
| BENIN | 629 | 5.72 | 0.11 | 0* | 2.22 | 1 |
| BURKINA FASO | 859 | 8.85 | 0.11 | 0* | 2.05 | 1 |
| BURUNDI | 337 | 9.97 | 0.04 | 0.3* | 2.25 | 1 |
| CAMEROON | 2,459 | 12.19 | 0.01* | 0.53 | 1.81 | 1 |
| CENTRAL AFRICAN REPUBLIC | 391 | 10.31 | 0* | 0.77 | 1.7 | 1 |
| CHAD | 1,620 | 7.43 | 0.13 | 0.31 | 2.68 | 0 |
| CIV | 1,061 | 6.56 | 0.11 | 0.85 | 2.92 | 0 |
| CONGO | 317 | 7.28 | 0.1 | 0.32 | 1.41 | 0 |
| DEMOCRATIC PEOPLE'S REPUBLIC OF KOREA | 308 | 0* | 0^[[2]](#footnote-2)^ | 0* | 0.64 | 2 |
| DEMOCRATIC REPUBLIC OF THE CONGO | 6,084 | 11.87 | 0.08 | 0.13* | 2.71 | 1 |
| EGYPT | 3,380 | 4.12 | 0.19 | 1.69 | 3.05 | 0 |
| ETHIOPIA | 3,342 | 8.21 | 0.01* | 0.09* | 1.16 | 2 |
| GHANA | 1387 | 9.7 | 0.08 | 0.44 | 2.54 | 0 |
| GUINEA | 1,661 | 8.03 | 0.11 | 1.03 | 2.91 | 0 |
| INDIA | 132,609 | 11.25 | 0.13 | 1.67 | 1.26 | 0 |
| INDONESIA | 4,577 | 9.86 | 0.25 | 2.86 | 1.22 | 0 |
| IRAN (ISLAMIC REPUBLIC OF) | 2,376 | 3.3 | 0* | 0.34 | 1.17 | 1 |
| IRAQ | 1,824 | 14.01 | 0.08 | 2.99 | 2.07 | 0 |
| JORDAN | 313 | 0.96* | 0* | 0* | 1.33 | 3 |
| KENYA | 1,669 | 12.32 | 0.07 | 0.79 | 1.49 | 0 |
| LEBANON | 299 | 13.33 | 0.31 | 2.78 | 0.99 | 0 |
| MADAGASCAR | 2,016 | 16.57 | 0.18 | 0.82 | 1.94 | 0 |
| MALAWI | 632 | 18.32 | 0.08 | 6.27 | 1.12 | 0 |
| MALI | 813 | 12.68 | 0* | 0.12* | 2.27 | 2 |
| MOZAMBIQUE | 1,160 | 11.62 | 0.01* | 7.96 | 2.65 | 1 |
| MYANMAR | 1,198 | 5.01 | 0.06 | 0* | 0.61 | 1 |
| NEPAL | 1,220 | 2.97* | 0.14 | 0.33 | 1.15 | 1 |
| NIGER | 1,270 | 15.65 | 0.03* | 0.4 | 6.02* | 2 |
| NIGERIA | 48,603 | 1.33* | 0.031 | 0.07* | 4.29* | 3 |
| PAKISTAN | 23,992 | 9.98 | 0.19 | 2.31 | 2.34 | 0 |
| RWANDA | 495 | 4.04 | 0.36 | 0* | 1.36 | 1 |
| SAUDI ARABIA | 803 | 1.12* | 0* | 0.12* | 0.8 | 3 |
| SENEGAL | 520 | 11.92 | 0.1 | 0* | 1.85 | 1 |
| SOMALIA | 942 | 1.7* | 0.22 | 0.11* | 7.05* | 3 |
| SOUTH AFRICA | 1,557 | 16.08 | 0.19 | 10.46 | 1.52 | 0 |
| SOUTH SUDAN | 1,042 | 8.37 | 0.02* | 0.19* | 6.41* | 3 |
| SUDAN | 1,515 | 1.87* | 0.06 | 1.06 | 1.26 | 1 |
| SYRIAN ARAB REPUBLIC | 903 | 11.07 | 0.15 | 3.48 | 1.3 | 0 |
| THAILAND | 625 | 24.44 | 0.19 | 7.01 | 0.68 | 0 |
| TOGO | 307 | 6.19 | 0.23 | 0* | 2.12 | 1 |
| UGANDA | 1,947 | 13.06 | 0* | 0.1* | 1.98 | 2 |
| UNITED REPUBLIC OF TANZANIA | 2,745 | 2.62* | 0.07 | 0* | 2.15 | 2 |
| YEMEN | 1,965 | 7.96 | 0.01* | 1.58 | 1.67 | 1 |
| ZAMBIA | 764 | 11.42 | 0.22 | 0* | 1.68 | 1 |
| ZIMBABWE | 587 | 4.88 | 0.18 | 2.42 | 1.57 | 0 |

**Table A3**: Population in districts, aggregated districts, and provinces, by WHO region.

| WHO Region | Unit | Min | 25^th^ Percentile | Median | 75^th^ Percentile | Max |
| --- | --- | --- | --- | --- | --- | --- |
| AFRO | Aggregated Districts | 122 | 257,754 | 337,105 | 448,011 | 2,222,686 |
|  | Districts | 122 | 44,826 | 78,435 | 135,163 | 2,043,145 |
|  | Provinces | 122 | 159,010 | 404,950 | 899,795 | 17,190,447 |
| EMRO | Aggregated Districts | 2,686 | 279,834 | 371,226 | 532,043 | 3,079,322 |
|  | Districts | 100 | 13,625 | 32,095 | 85,345 | 3,079,322 |
|  | Provinces | 857 | 127,359 | 301,717 | 688,033 | 43,049,154 |
| SEARO | Aggregated Districts | 1,459 | 303,604 | 458,883 | 731,658 | 4,372,218 |
|  | Districts | 117 | 19,906 | 52,055 | 269,771 | 4,372,218 |
|  | Provinces | 2,325 | 90,743 | 314,125 | 1,137,624 | 76,365,966 |
| WPRO | Aggregated Districts | 221 | 242,614 | 332,234 | 458,452 | 4,140,229 |
|  | Districts | 221 | 17,359 | 32,804 | 49,696 | 4,140,229 |
|  | Provinces | 12,418 | 187,426 | 285,523 | 434,091 | 5,564,043 |

**Figure A2**: Distribution of Indicators Across all Countries by WHO Region (n=79), 2015-2017.


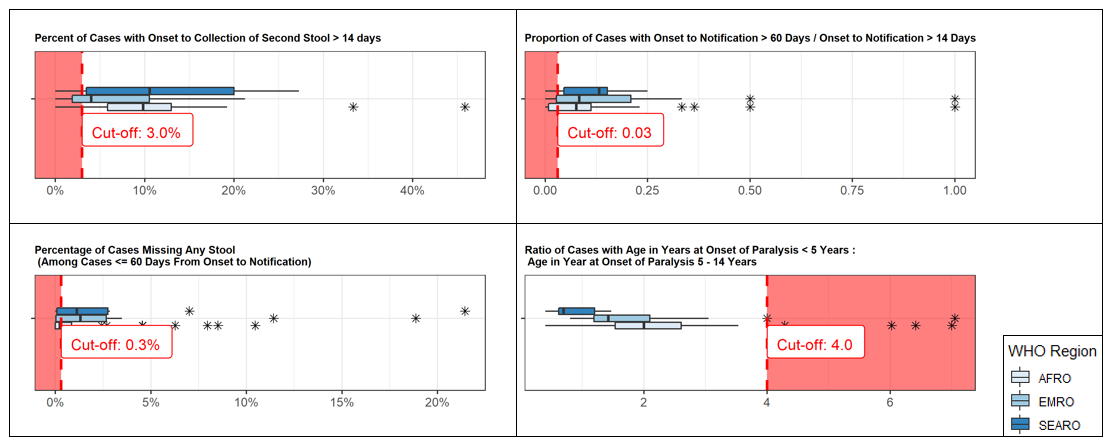


1. ^49 countries met the inclusion criteria of ≥ 250 AFP cases reported from 2015 – 2017 (excluding Eritrea and Morocco).^

   ^4 There were 0 cases that had an onset to notification of >14 days. This number serves as the denominator for the proportion, and was thus not flagged.^

   * ^Signifies meeting the criteria to be flagged for this indicator.^  [↑](#footnote-ref-1)
2. [↑](#footnote-ref-2)
